# The protocadherin-15-LHFPL5 tip link complex is a heterotetrameric assembly in hair cell stereocilia

**DOI:** 10.1016/j.bpj.2026.02.003

**Published:** 2026-02-10

**Authors:** Sarah Clark, Jaba Mitra, Johannes Elferich, April Goehring, Jingpeng Ge, Taekjip Ha, Eric Gouaux

**Affiliations:** 1 Vollum Institute, Oregon Health & Science University, Portland, Oregon; 2 Department of Materials Science and Engineering, University of Illinois Urbana-Champaign, Champaign, Illinois; 3 Department of Biophysics and Biophysical Chemistry, Johns Hopkins University, Baltimore, Maryland; 4 Howard Hughes Medical Institute, Oregon Health & Science University, Portland, Oregon

## Abstract

Hearing and balance rely on the conversion of a mechanical stimulus into an electrical signal, a process known as mechanosensory transduction (MT). In vertebrates, this process is accomplished by a MT complex located in hair cells of the inner ear. While the past three decades of research have identified many subunits that are important for MT and revealed interactions between these subunits, the composition and organization of a functional complex remain unknown. The major challenge associated with studying the MT complex is its extremely low abundance in hair cells; current estimates of MT complex quantity range from 3 to 60 amol per cochlea or utricle, well below the detection limit of most biochemical assays used to characterize macromolecular complexes. Here we describe the optimization of two single-molecule assays, single-molecule pull-down (SiMPull), and single-molecule array (SiMoA), to study the composition and quantity of the native MT subunits, protocadherin-15 (PCDH15) and lipoma HMGIC fusion partner-like protein 5 (LHFPL5). We demonstrate that these assays are capable of detecting and quantifying amol quantities of native protein derived from mouse cochlea and utricle. Our results illuminate the stoichiometry of PCDH15- and LHFPL5-containing complexes and establish SiMPull and SiMoA as productive methods for probing the abundance, composition, and arrangement of subunits in the native MT complex.

## Significance

In this work, the authors develop and employ single-molecule methods to detect, define the stoichiometry, and quantitate amol quantities of the hair cell mechanosensory transduction complex.

## Introduction

Hearing and balance are critical for everyday life, yet the molecular basis of these sensations remains largely unresolved. This is due, in part, to challenges associated with studying the mechanosensory transduction (MT) complex, the protein machinery responsible for converting the mechanical stimulus associated with a sound or fluid movement into an electrical signal that is processed by the brain (1). Numerous genetic and biochemical studies have identified MT subunits important for MT: the tip-link proteins, protocadherin-15 (PCDH15) and cadherin-23 (CDH23), connect adjacent stereocilia and transduce force associated with stereocilia movement into ion channel opening (2,3), while transmembrane channel-like proteins 1 and 2 (TMC1/2) are the likely pore-forming subunits of the ion channel and are located at the lower end of the tip-link (4,5,6). The lipoma HMGIC fusion partner-like protein 5 (LHFPL5, also known as TMHS) (7,8,9), transmembrane inner ear protein (TMIE) (10,11,12), and calcium and integrin-binding proteins 2 and 3 (CIB2/3) (13,14) are predicted to assemble with TMC1/2 and PCDH15 to form a functional MT complex. Additional proteins, including transmembrane O-methyltransferase (TOMT) (15,16), whirlin (17,18), and myosin XVa (19,20), also play an important role in hearing and possibly interact with MT subunits at different stages of complex assembly.

Structural and biochemical studies have shed light on interactions between some of these subunits and their probable organization in the vertebrate MT complex. Recombinantly expressed PCDH15 binds to LHFPL5 and CDH23, and their respective protein complexes have been characterized by cryo-electron microscopy (9) and x-ray crystallography (21). Peptides of TMC1 form a complex with CIB2 and CIB3 (14,22), and mutagenesis experiments in mouse hair cells suggest that TMIE directly interacts with TMC1 (12). In accordance with these studies, the recently elucidated structures of the native *C. elegans* TMC-1 and TMC-2 complexes revealed a dimeric complex that is composed of two copies of TMC-1/2, TMIE, and CIB2/3 (23,24), the latter of which is known as CALM-1 in *C. elegans*. Further, while numerous immunoprecipitation and yeast two-hybrid experiments have suggested interactions between MT subunits (10,12,15,25,26), these experiments have been hampered by the inability to express biochemically well-behaved TMC1 and TMC2 proteins. TMC1 and TMC2 do not migrate to the plasma membrane in heterologous cell lines (27), preventing reconstitution of the vertebrate MT complex. To elucidate the composition and organization of the vertebrate MT complex, it is therefore important to use native tissue.

A well-known hurdle in studying the vertebrate MT complex is its extremely low abundance in cochlea and utricle tissue. Electrophysiological experiments of mouse hair cells suggest that there are approximately two functional MT channels per stereocilium (28,29,30), which amounts to about 3 amol of MT complex per mouse cochlea. High-resolution imaging methods also suggest that the complex is present in amol quantities, but the precise number of complexes varies depending on experimental parameters. On the one hand, freeze-etch electron microscopy images of stereocilia tips from bullfrogs and guinea pigs indicate that there is only one tip-link per stereocilia (31), while on the other hand, photobleaching experiments report an average of 7.1 TMC1 molecules per stereocilia in the inner hair cells of mice aged postnatal day 4 (P4) (32). Moreover, electron tomography images of labeled PCDH15 on native stereocilia derived from mice at ages P6–P9 indicate there are anywhere from 2 to more than 5, PCDH15 dimers per tip, plus many more PCDH15 molecules that are localized on the stereocilia shaft as lateral links (33). Attomole quantities of protein cannot be studied using conventional biochemical methods such as western blots, mass spectrometry, or enzyme-linked immunosorbent assays (ELISAs), all of which require fmol, or more, of material. Ultrasensitive ELISA (34) and immuno-PCR (35,36) techniques developed in recent years can detect low amol quantities of protein, but these assays are limited in their ability to accurately measure protein quantity or assess interactions between proteins and their stoichiometry. Given the limitations of current methodologies, we sought to establish highly sensitive methods that can be used to study the native MT complex.

Single-molecule techniques offer many advantages in the study of low-abundance macromolecular complexes (37) because they allow for the capture of small amounts of material directly from tissue extracts. Here, we describe the optimization of two single-molecule assays, single-molecule pull-down (SiMPull) (37) and single-molecule array (SiMoA) (38), to study the composition and quantity of the native MT complex subunits PCDH15 and LHFPL5 from solubilized mouse cochlea and utricle tissue. We demonstrate that SiMPull and SiMoA assays are capable of detecting and quantifying single-digit amol of protein. Our results shed light on the organization of the native PCDH15/LHFPL5 complex and establish techniques to characterize and quantify extremely low-abundance, multisubunit complexes.

## Materials and methods

### Construct design

**Figure 1:**
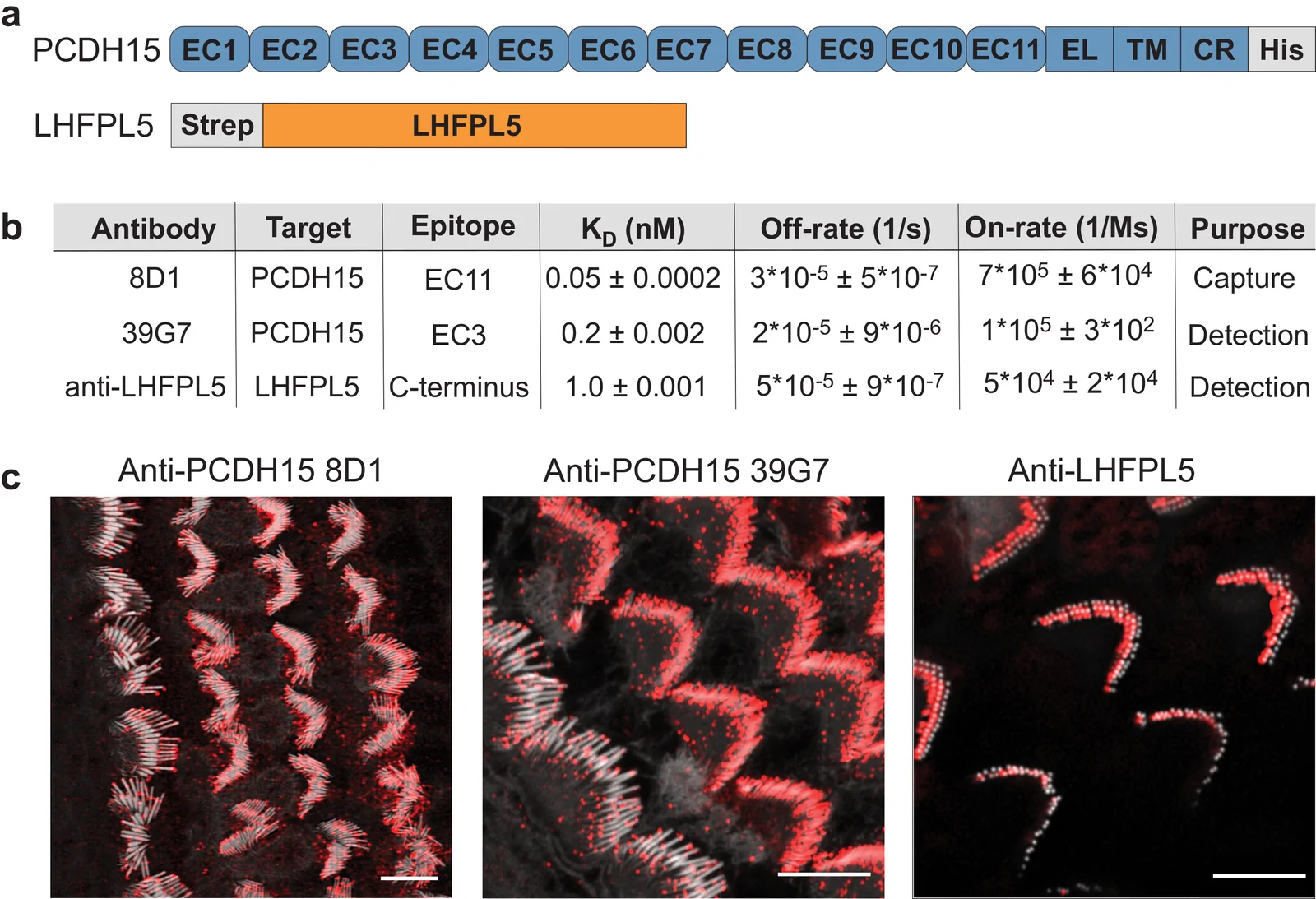
Characterization of reagents used for SiMPull and SiMoA assay development. (*a*) Schematics of the recombinant PCDH15 and LHFPL5 constructs. EC, extracellular cadherin domains; EL, extracellular linker domain; TM, transmembrane domain; CR, cytoplasmic region; His, 8xHis tag; Strep, twin-strep tag. (*b*) Table of antibody binding constants, off-rates, and on-rates, measured by biolayer interferometry. Binding constants shown were measured using intact antibodies. (*c*) Immunostaining of WT cochlea with anti-PCDH15 and anti-LHFPL5 antibodies. Actin was stained with SirActin-405, shown in white, and mouse and rabbit monoclonal antibodies were detected with an anti-mouse-Alexa594 or anti-rabbit-Alexa594 secondary antibody, shown in red. Scale bars, 5 *μ*m. Note: the anti-PCDH15 8D1 image was acquired at lower magnification compared with the other panels. For all antibodies, multiple immunostaining experiments were performed on different days using varying imaging conditions. All results were consistent, and the clearest representative image for 8D1 was selected for presentation here.

The PCDH15 and LHFPL5 constructs correspond to the canonical *Mus musculus* sequences as recorded in the UniProt database (Uniprot: Q99PJ1, Q4KL25). The PCDH15 constructs were synthesized by GenScript and the LHFPL5 construct is the same construct used in previous studies (residues 2–219) (9). The PCDH15 construct used for SiMPull and SiMoA experiments was composed of the full extracellular domain (EC1–EC11) and part of the cytoplasmic region (CR), residues 1–1462, and is referred to as PCDH15. The PCDH15 construct used for generation of the 8D1 antibody was composed of the EC11-CR domains, residues 1–30 and 1145–1462, and is referred to as PCDH15_1EC_. All constructs were cloned into the pEG BacMam (39) vector under control of the CMV promoter, allowing expression by infection using baculovirus produced in Sf9 cells. Schematics of the sequences, fluorophores, and affinity tags are shown in Fig. 1.

### Expression and purification of PCDH15 in complex with LHFPL5

The recombinant PCDH15/LHFPL5 and PCDH15_1EC_/LHFPL5 complexes were expressed and purified using the protocol described in Ge et al. (9) The protocol was identical for both protein complexes. Briefly, HEK293 tsa201 cells were co-infected with PCDH15 and LHFPL5 BacMam viruses at a MOI of 1:1. Cultures were supplemented with 10 mM sodium butyrate 12 h postinfection and transferred to 30°C. Cells were harvested 48 h postinfection and lysed in buffer containing 100 mM Tris (pH 8.0), 150 mM NaCl, 1% (w/v) digitonin and protease inhibitors for 2 h at 4°C. The solubilized material was incubated with Strep-Tactin resin, washed with buffer A containing 20 mM Tris (pH 8.0), 150 mM NaCl, 0.07% (w/v) digitonin and eluted with buffer A plus 5 mM desthiobiotin to remove free His-tagged PCDH15 or PCDH15_1EC_. The elution was then incubated with TALON resin, washed with buffer A plus 10 mM imidazole to remove free strep-tagged LHFPL5, and further eluted with buffer A plus 200 mM imidazole. The complex was then further purified by size-exclusion chromatography in buffer A. Peak fractions were collected and analyzed by SDS-PAGE.

### Generation, expression, and purification of antibody fragments

The anti-PCDH15 8D1 antibody was raised by D. Cawley through the OHSU Vaccine & Gene Therapy Institute. Liposomes containing asolectin/cholesterol/lipidA/brain polar lipid (60:17:3:20) were extruded through a 200-nm filter. The PCDH15_1EC_/LHFPL5 antigen was reconstituted into the liposomes by first saturating the liposomes with 5 mM *n*-dodecyl-β-D-maltoside, then adding purified antigen at a protein/lipid ratio of 1:50 (w/w). Detergent was removed by three additions of 250 mg of biobeads. For the first two additions, the biobeads were incubated for 2 h, and the final incubation was overnight. Mice were immunized with 25 *μ*g of proteoliposomes following a standard schedule. Hybridoma cell lines were generated as described (40) and hybridoma supernatants were screened by ELISA using PCDH15_1EC_/LHFPL5 complex as the antigen to identify clones. Positive clones were further screened by fluorescence size-detection chromatography (FSEC) (41) and western blotting. The clone 8D1 was selected and the monoclonal antibody was produced through hybridoma cell culture. The anti-LHFPL5 monoclonal antibody was purchased from Abcam (Cambridge, UK, catalog no. ab232650). The anti-PCDH15 39G7 monoclonal antibody was generated using standard techniques by GenScript using the soluble PCDH15 EC1-EL extracellular domain as an antigen, as described in Elferich et al. (33).

The amino acid sequence of the variable domain of 39G7 was provided by GenScript as a part of the antibody generation service. The amino acid sequence of the variable domain of the anti-LHFPL5 mAb was determined by mass spectrometry and the construct was synthesized by GenScript. The DNA sequences encoding the heavy and light chains of the variable domains of the 39G7 and anti-LHFPL5 antibodies were cloned into the pEG BacMam vector for baculovirus expression in HEK293 tsa201 cells. A GFP tag and 8xHis tag were added to the C-terminus of the heavy chain in both constructs. Cells were infected with 39G7 or anti-LHFPL5 BacMam viruses at a MOI of 1:1. Cultures were supplemented with 10 mM sodium butyrate 12 h postinfection and transferred to 30°C. Cells were harvested 96 h postinfection and the cell medium was filtered and concentrated to 200 mL. Concentrated medium was incubated with TALON resin, washed with TBS plus 10 mM imidazole, and eluted with TBS plus 200 mM imidazole. The antibody fragments were further purified using size-exclusion chromatography in phosphate-buffered saline (PBS).

### Characterization of antibodies

To determine the epitope of the 8D1 and 39G7 antibodies, different constructs of YFP-tagged PCDH15 were generated that each lacked an EC domain. These constructs were as follows: EC11-EL, EC10-EL, EC9-EL, EL-EC8, EL-EC7, EL-EC6, EC5-EL, EC4-EL, EC3-EL, EC2-EL, and EC1-EL. The constructs were transiently transfected into HEK293 cells, harvested after ∼96 h, and the cell medium was screened against the antibodies using FSEC. The anti-LHFPL5 antibody is listed as targeting a C-terminal epitope on the Abcam website.

The affinities of the 8D1, 39G7, and anti-LHFPL5 monoclonal antibodies for their antigen were determined with biolayer interferometry using an OctetRED384 instrument. Fab affinities were not determined. Experiments were performed at 20°C and assay buffer was composed of TBS plus 0.07% (w/v) digitonin. Anti-mIgG Fc Capture (AMC) biosensors were prehydrated for 10 min in assay buffer before use. The recombinant PCDH15/LHFPL5 complex was used as an antigen. Antibodies and PCDH15/LHFPL5 antigen were diluted into assay buffer immediately before use and 100 *μ*L of each sample was loaded into 384-well microplates. Antibodies were diluted to 25 *μ*g/mL and the concentration of PCDH15 EC11-EL ranged from 0.75 to 220 nM. Each concentration was performed in duplicate and a range of concentrations were used to increase accuracy of affinity calculations. The OctetRED method was set as follows: equilibration (180 s), precondition (5 s), equilibration 2 (60 s), antibody loading (600 s), baseline (300 s), association of PCDH15 antigen (900 s), and dissociation (1800 s). Data were analyzed using the Octet Data Analysis software by fitting the association and dissociation curves. A blank sample consisting of 0 nM PCDH15/LHFPL5 was used as a reference during processing.

### Immunofluorescence

Cochlea immunostaining experiments were performed as described in Elferich et al. (33). Cochlea were dissected from mice at ages P6–P9 in DMEM/F12 medium from Gibco (Waltham, Massachusetts). The tissue was incubated for 30 min in 10 *μ*g/mL of the indicated antibody in PBS without calcium or magnesium, followed by washing in the same buffer. The tissue was next fixed in a buffer composed of 4% paraformaldehyde in PBS for 10 min. After three washes in PBS, the tissue was permeabilized and blocked using PBS with 0.1% Triton X-100, 5% bovine serum albumin (BSA), and 10% standard goat serum. Subsequently, the tissue was stained with goat-anti-rabbit or goat-anti-mouse antibodies fused to Alexa-594 and phalloidin fused to Alexa-405. After three washes with PBS, the tissue was mounted with Vectashield mounting medium and imaged using a Zeiss LSM 980 confocal microscope using a 63×/1.49 NA objective.

### Cochlea and utricle solubilization

Cochlea and utricle samples were prepared by homogenizing cochlea or utricles from P6 mice on ice with a pestle homogenizer in lysis buffer consisting of 1% C12M, 0.2% CHS, 50 mM Tris, 40 mM NaCl, 10 mM KCl, 1 mM EDTA, 1 mM protease inhibitors (0.8 *μ*M aprotinin, 2 *μ*g/mL leupeptin, and 2 *μ*M pepstatin), and 0.2 mg/mL BSA (pH 8.0). Lysis buffer (20 *μ*L) was added to each cochlea. Four cochleas were pooled per SiMPull experiment and 2–6 cochleas or utricles were pooled per SiMoA experiment. Homogenized cochleas were incubated for 1 h at 4°C and insoluble material was pelleted by centrifuging for 10 min at 14,000 rpm. The supernatant was collected and used immediately for SiMPull or SiMoA experiments.

### SiMPull

Coverslips and glass slides were prepared as described in Jain et al. (42). In brief, the coverslips and glass slides were extensively cleaned, passivated, and coated with methoxy polyethylene glycol and 2% biotinylated PEG. A flow chamber was created by drilling 0.75-mm holes in the glass slide and placing double-sided tape between the holes. A coverslip was placed on top of the slide and the edges were sealed with epoxy, creating tiny flow chambers. Streptavidin (0.25 mg/mL) was then applied to the slide, allowed to incubate for 5 min, and washed off with T50 BSA buffer consisting of 50 mM Tris (pH 8.0), 50 mM NaCl, 0.25 mg/mL BSA. Anti-PCDH15 8D1 capture antibody was biotinylated using NHS-PEG4-biotin (Thermo Fisher, CAS no. 21330) and anti-LHFPL5 antibody was labeled with Alexa647 using a Zip Alexa Fluor Rapid Antibody Labeling Kit from Thermo Fisher (Waltham, Massachussetts, CAS no. Z11235). Excess dye and biotin were removed using two Zeba spin desalting columns per labeling reaction and labeling was confirmed using FSEC (39). The ratio of LHFPL5 antibody to Alexa647 dye was determined using the “Proteins and Labels” application in a Nanodrop spectrophotometer and ranged from 4 to 6 fluorophores per antibody. Biotinylated 8D1 at approximately 7 *μ*g/mL was applied to the slide, allowed to incubate for 10 min, and washed off with buffer B containing 0.005% C12M, 0.001% CHS, 50 mM Tris, 40 mM NaCl, 10 mM KCl, 0.1 mM EDTA, and 0.2 mg/mL BSA (pH 8.0). For colocalization and photobleaching experiments of the recombinant PCDH15/LHFPL5 complex, the sample was diluted to 100 pM in buffer B and incubated in the chamber for 5 min. For cochlea supernatant, the sample was applied to the chamber in 10-*μ*L increments, with 5 min incubation per application. Following sample application, the chamber was washed and 3 *μ*g/mL fluorophore-labeled detection antibody was applied to the chamber for 5 min. The chamber was imaged immediately after the final wash step using the total internal reflection microscopy (TIRF) module of a Leica DMi8 microscope with a 100× oil-immersion objective. Fifteen images were collected at different locations per chamber using an Andor iXon Ultra 888 EMCCD camera with a 13-*μ*m pixel size, frame rate of 26 frames/s, 300 ms exposure time, and EM gain set at 1000. At least two biological replicates were performed per sample and the two-tailed unpaired Student’s *t*-test was used to assess statistical significance. Photobleaching movies were collected by exposing the imaging area for 3 min.

Two negative control samples were used in SiMPull experiments. The first negative control sample lacks the 8D1 capture antibody but is otherwise identical to the measured sample, which enables counting of nonspecific binding events. The second negative control is cochlea solubilized from PCDH15^av−3J^ mice (43), in which the PCDH15 gene bears a frameshift mutation and a premature stop codon. The cochlea and utricles for PCDH15^av−3J^ mice were dissected at P6 and were solubilized exactly as described for wild-type (WT) tissue.

Molecule quantitation and colocalization were determined using the ComDet v0.5.5 plugin for FIJI (44) available at (https://github.com/UU-cellbiology/ComDet). First, a 45 × 45 *μ*m square was drawn in the center of the image to account for the decrease in fluorescence intensity at the periphery of the image. Only puncta that fell within the square were counted. Next, ComDet was run using the following detection parameters: particle size = 3.4 pixels; intensity threshold = 3.4 standard deviations; ROI shape = oval. For colocalization analysis, the maximum distance between colocalized spots was 3 pixels.

Photobleaching movies were analyzed using a custom python script that is available on Zenodo (https://zenodo.org/records/8161179). This python script plots the decrease in intensity vs. time for selected puncta in a photobleaching movie. The user first selects puncta based on user-defined size and intensity thresholds. The intensity of the selected puncta are then plotted against the duration of the photobleaching movie. The user manually scores each trace for the number of photobleaching steps (1-step, 2-step, 3-step, 4-step, etc.) based on the presence of a sharp decrease in intensity. To verify scoring, the intensity of the molecules scored as bleaching in one or two steps was also taken into account; on average we expect the fraction of molecules bleaching in two steps to be twice as bright as molecules bleaching in one step.

### SiMoA

Recombinant PCDH15/LHFPL5 complex and cochlea samples were quantitated using the Quanterix homebrew assay kit and a Quanterix SR-X Biomarker Detection System. Anti-PCDH15 8D1 was conjugated to paramagnetic beads for capturing analyte and anti-PCDH15 39G7 mAb or anti-LHFPL5 mAb was biotinylated for analyte detection according to instructions in the homebrew assay kit. Cochlea and utricles samples were solubilized as described above and 2–6 cochlea were pooled per sample. The 2-step method was employed wherein beads, sample, and detection antibody were incubated simultaneously for 30 min with continuous shaking at room temperature. Sample binding buffer consisted of 1% C12M, 0.2% CHS, 1% casein in PBS, 1 mM EDTA, protease inhibitors, and 10 *μ*g/mL mouse IgG. After incubation, the beads were washed with buffer C containing 0.005% C12M in PBS. Streptavidin β-galactosidase (120 pM) was applied for 7 min and beads were washed again with buffer C, followed by a short wash with SiMoA wash buffer B (buffer composition is proprietary), and allowed to dry for 10 min. Dried plates were then placed in the SR-X for substrate application and analysis. All samples were run in duplicate and at least two biological replicates were performed per sample. The negative controls for SiMoA experiments were cochlea or utricles dissected from PCDH15^av−3J^ mice that were prepared identically to WT samples.

## Results

### Development of an ultrasensitive SiMPull assay using recombinant PCDH15/LHFPL5

To examine individual PCDH15- and LHFPL5-containing complexes from cell or tissue extracts (37,42), we employed SiMPull, a technique that combines a conventional pull-down assay with TIRF. The first step of this assay is to capture PCDH15- and LHFPL5-containing complexes from a cell extract using a monoclonal antibody (mAb) immobilized on a coverslip. Next, a mAb or antibody fragment (Fab) linked to a fluorophore is used to detect the complex. Subunit interactions are assessed through colocalization experiments, wherein multiple mAbs/Fabs are labeled with different fluorophores and applied to the slide simultaneously. Subunit stoichiometry is measured by labeling a mAb/Fab with a single GFP or YFP molecule and photobleaching the imaging area. High-affinity antibodies are therefore a key component of a successful SiMPull assay. We generated antibodies directed against different regions of PCDH15 by immunizing mice and rabbits with constructs of recombinantly expressed PCDH15 and the PCDH15/LHFPL5 complex (Fig. 1 *a*). We obtained two antibodies with exceptional binding kinetics that recognize native PCDH15: 8D1, which binds to the EC11 domain of PCDH15, and 39G7, which recognizes the EC3 domain of PCDH15. 8D1 and 39G7 both bind PCDH15 with a subnanomolar K_D_ (Figs. 1 *b* and S1) and 8D1 exhibits a remarkably fast on rate of 7 × 10^5^ M^−1^ s^−1^. Immunostaining of WT mouse cochlea with these antibodies produces robust fluorescence that is localized to stereocilia tips (Fig. 1 *c*). The 39G7 mAb was recently employed in cryoelectron tomography experiments to elucidate the molecular structures and conformational states of PCDH15 on stereocilia (33). Further, we characterized an anti-LHFPL5 antibody and found that it binds to LHFPL5 with ∼1 nM affinity and similarly stains the tips of stereocilia in WT mice (Fig. 1, *b* and *c*). Determination of the amino acid sequence of the variable domains of these three antibodies allowed us to generate recombinantly expressed Fab constructs, protein constructs that facilitate single-molecule photobleaching experiments.

**Figure 2:**
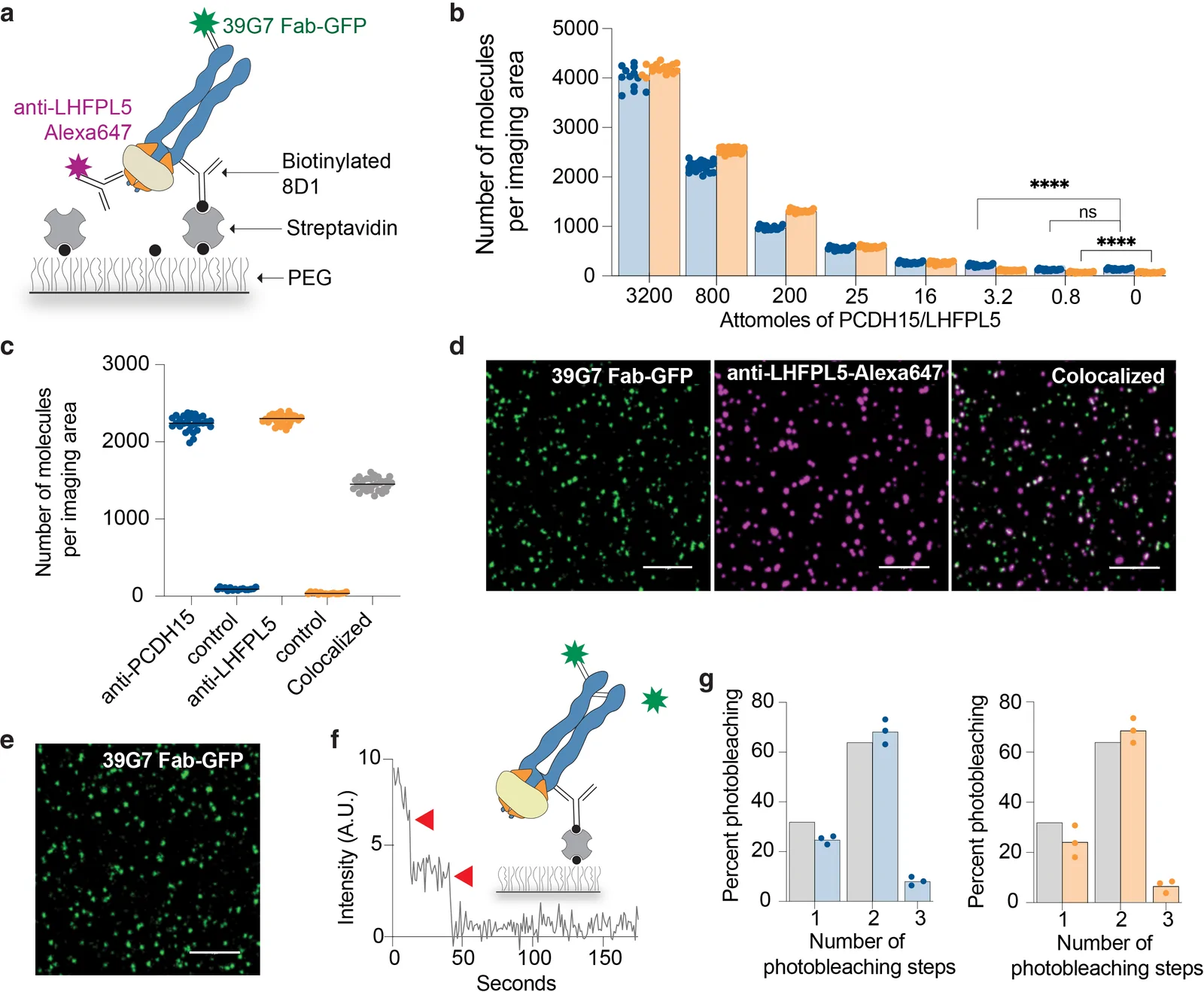
SiMPull assay development with recombinant PCDH15/LHFPL5. (*a*) Schematic depiction of the SiMPull experiment. PCDH15/LHFPL5 complexes are immobilized on a passivated coverslip coated with anti-PCDH15 mAb 8D1. PCDH15 (*blue*) and LHFPL5 (*orange*) are detected with Fab-GFP or mAbs conjugated to Alexa dyes. (*b*) SiMPull dilution analysis of PCDH15/LHFPL5. PCDH15 was detected with 39G7 Fab-GFP and LHFPL5 was detected with anti-LHFPL5 mAb conjugated to Alexa647 (^∗∗∗∗^*p* < 0.0001 calculated using a two-tailed unpaired Student’s *t*-test). The imaging area used for analysis is a 45 × 45 *μ*m square drawn in the center of the 133.12 × 133.12 *μ*m image. (*c*) Total number of recombinant PCDH15, LHFPL5, and colocalized PCDH15/LHFPL5 molecules are shown. Control samples were prepared identically but without the 8D1 capture antibody to assess nonspecific binding. We observed 65% of PCDH15 molecules colocalized with LHFPL5 molecules. *N* = 30 images analyzed over 2 independent experiments. The imaging area used for analysis is a 45 × 45 *μ*m square drawn in the center of the 133.12 × 133.12 *μ*m image. (*d*) Representative TIRF images of recombinant PCDH15/LHFPL5 complex detected with anti-PCDH15 Fab 39G7-GFP and anti-LHFPL5 mAb. Scale bars, 5 *μ*m. (*e*) Representative TIRF image of PCDH15 detected with the 39G7 Fab-GFP. (*f*) Representative trace showing two step photobleaching (*red arrows*) of the 39G7 Fab-GFP. Inset is a schematic of the recombinant PCDH15/LHFPL5 complex detected with 39G7 Fab-GFP. (*g*) Summary of photobleaching step distribution for PCDH15/LHFPL5 detected with 39G7 Fab-GFP (*blue bars*) or anti-LHFPL5 Fab-GFP (*orange bars*). The photobleaching step distributions correspond to a binomial distribution that assumes a dimeric protein and 80% GFP maturation (*gray bars*). *N* = 600 spots were analyzed from 3 photobleaching movies (200 spots/movie) collected from 2 independent experiments.

To develop a SiMPull assay of sufficient sensitivity to detect amol quantities of PCDH15 and LHFPL5, we used recombinantly expressed the PCDH15/LHFPL5 complex as a control. The PCDH15 construct included the entire extracellular cadherin domain and extended into the CR (residues 1–1462) and the LHFPL5 construct encompassed the full-length protein (Fig 1 *a*). Multiple antibody combinations were assessed for capture and detection, allowing us to determine that 8D1 is the best antibody for capturing protein from low concentration samples, likely due to its fast on rate and slow dissociation rate (Fig. S1 *a*). Passivated slides were coated with biotinylated 8D1 to capture PCDH15/LHFPL5, which was then detected with an anti-PCDH15 39G7 Fab fused to GFP (39G7 Fab-GFP) or with an anti-LHFPL5 mAb conjugated to Alexa647 (Fig. 2 *a*). The assembled immunocomplexes appear as a fluorescent puncta upon imaging with TIRF (Fig 2), with each puncta representing an individual PCDH15/LHFPL5 complex. We quantitated the number of recombinant PCDH15/LHFPL5 puncta at different concentrations and compared these values with a negative control sample that lacked the capture antibody to determine the limits of detection. We routinely detected 3.2 amol of PCDH15 and 0.8 amol of LHFPL5 with signal/noise about fivefold above background (Fig. 2 *b*), indicating that the assay is adequately sensitive to detect native PCDH15 and LHFPL5. The difference in sensitivity between PCDH15 and LHFPL5 is likely due to the increased brightness of the Alexa647 fluorophore relative to a GFP molecule. For comparison, we performed a western blot with the same quantity of protein and discovered that we can detect only 12 pmol of LHFPL5 with the anti-LHFPL5 mAb (Fig. S2). 8D1 and 39G7 both recognize a folded, three-dimensional epitope and are unsuitable for western blot.

Next, we analyzed the composition and stoichiometry of the recombinantly expressed PCDH15/LHFPL5 complex to confirm it forms a heteromeric complex, as expected from structural studies (9). Protein colocalization is determined by labeling the antibodies that target each protein with different fluorophores, in our case GFP and Alexa647, and then measuring how many of the fluorescent puncta overlap. Overlapping puncta indicates that the antibodies, which each target a different subunit, are labeling proteins in the same complex. Application of 10 *μ*L of 100 pM PCDH15/LHFPL5 complex to the sample chamber, followed by simultaneous application of the 39G7 Fab-GFP and anti-LHFPL5-Alexa647 mAb, enabled assessment of the degree of colocalization between PCDH15 and LHFPL5. The recombinant LHFPL5 puncta colocalized with PCDH15 puncta 62% of the time (Fig. 2, *c* and *d*), consistent with the expectation the two proteins are found in the same complex. Although PCDH15 and LHFPL5 form a stable, homogenous complex, their lack of 100% colocalization can be attributed to a number of factors, including incomplete antibody binding, incomplete fluorophore labeling, and dissociation of the PCDH15/LHFPL5 complex.

To determine the stoichiometry of PCDH15 and LHFPL5, we captured the complex with 8D1 and, in separate experiments, labeled PCDH15 with 39G7 Fab-GFP and labeled LHFPL5 with the anti-LHFPL5 Fab-GFP. The imaging area was photobleached for 3 min and the resulting movies were analyzed to determine stoichiometry. The number of photobleaching steps, or distinct decreases in fluorescent intensity, that a molecule undergoes indicates the number of fluorophores that are present in that puncta. For example, a dimeric protein that is labeled with two GFP-tagged Fabs will exhibit two photobleaching steps. Approximately 68% of 39G7 Fab-GFP molecules and 68% of anti-LHFPL5 Fab-GFP molecules bleached in two steps, demonstrating that PCDH15 and LHFPL5 are present in two copies each (Fig. 2, *e*–*g*). The single-step photobleaching events can be attributed to incomplete maturation of the GFP-tagged Fab, as demonstrated by comparing the photobleaching step distribution with a binomial distribution that assumes a dimeric protein and 80% GFP maturation (Fig. 2 *g*, *gray bars*). The small portion of three step bleaching events might be due to a combination of overlapping complexes or to nonspecific Fab binding. These results demonstrate that we have developed a highly sensitive, robust assay capable of detecting and defining the stoichiometry of native PCDH15- and LHFPL5-containing complexes.

### Native PCDH15 and LHFPL5 form a heterotetrametric complex

**Figure 3:**
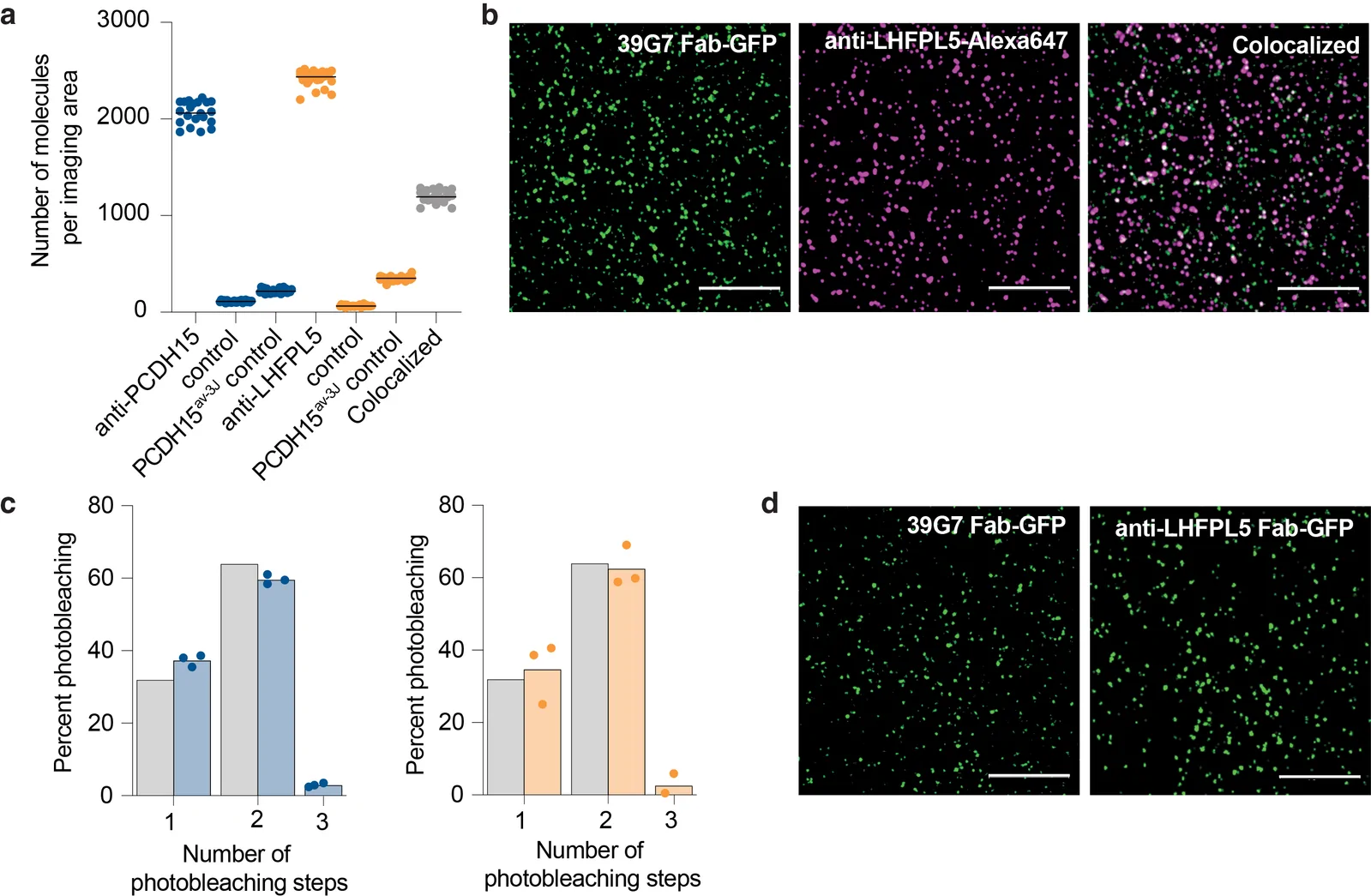
Native PCDH15/LHFPL5 form a heterotetrameric complex. (*a*) Observed colocalization of native PCDH15 with native LHFPL5 was 58%. Negative controls included an identically prepared sample without 8D1 capture antibody to assess nonspecific binding and cochlea from PCDH15^av−3J^ cochlea. *N* = 25 images analyzed over 2 independent experiments. The imaging area used for analysis is a 45 × 45 *μ*m square drawn in the center of the 133.12 × 133.12 *μ*m image. (*b*) Representative TIRF images of recombinant PCDH15/LHFPL5 complex detected with anti-PCDH15 Fab 39G7-GFP and anti-LHFPL5 mAb. Scale bars, 5 *μ*m. (*c*) Summary of photobleaching step distribution of native PCDH15 (*blue bars*) and native LHFPL5 (*orange bars*). The photobleaching step distributions correspond well with a binominal distribution (*gray bars*) that assumes a dimeric protein and 80% GFP maturation. *N* = 600 spots were analyzed from 3 photobleaching movies (200 spots/movie) collected from 2 independent experiments. (*d*) Representative TIRF images of PCDH15 detected with the 39G7 Fab-GFP and LHFPL5 detected with anti-LHFPL5 Fab-GFP. Scale bars, 5 *μ*m.

While it is suspected that native PCDH15 forms a complex with LHFPL5 based on data from biochemical experiments and mutagenesis studies in mice (7,9), the interaction between native PCDH15 and LHFPL5 has never been demonstrated and it is unclear what portion of the PCDH15 population associates with LHFPL5. To address these questions and demonstrate the utility of the SiMPull assay in detecting native MT subunits, we pulled down native PCDH15-containing complexes from mouse cochlea. Cochlea were homogenized in a buffer supplemented with a nonionic detergent and the supernatant was applied to slides coated in the 8D1 antibody. Simultaneous application of the 39G7 Fab-GFP and anti-LHFPL5-Alexa647 mAb allowed us to measure colocalization between PCDH15 and LHFPL5. Approximately 58% of LHFPL5 subunits colocalized with PCDH15, indicating that the majority of PCDH15 molecules are in complex with LHFPL5 (Figs. 3, *a*, *b* and S3, *a*, *b*). Importantly, we did not observe a statistically significant signal above background for either PCDH15 or LHFPL5 when supernatant derived from the cochlea of PCDH15^av−3J^ mice (43), a mouse line in which the PCDH15 gene bears a frameshift mutation, were applied to the slide. We similarly performed photobleaching experiments to assess the stoichiometry of native PCDH15 and LHFPL5. Approximately 59% of 39G7 Fab-GFP molecules and 63% of LHFPL5 Fab-GFP molecules bleached in two steps, indicating that both proteins are present in two copies (Figs. 3, *c*, *d* and S3, *c*, *d*). Our results indicate that PCDH15 and LHFPL5 form a heterotetrameric complex in hair cells and demonstrate that the SiMPull assay is sufficiently sensitive to probe the assembly and stoichiometry of native MT subunits from solubilized mouse cochlea.

### SiMoA-based quantitation of native PCDH15 and LHFPL5

All of the approaches used to estimate the number of MT complexes relied on counting labeled subunits in images of stereocilia (31,32,33). While these methods have provided insight into the abundance of MT subunits, they are complicated by damage to the stereocilia during sample preparation and background staining of the labeled antibody. These methods are also labor intensive, requiring acquisition of dozens of high-resolution images. While SiMPull can be used to estimate the number of MT subunits, its accuracy is limited, in part due to the sample application method. The small volume of the SiMPull sample chamber requires iterative applications of the homogenized cochlea sample, introducing room for error. Additionally, due to the way that SiMPull chambers are constructed, each chamber varies slightly in volume capacity, ranging from 7 to 10 *μ*L. This variability in chamber volume is likely why the data from dilution experiments of recombinant PCDH15/LHFPL5 were not ideally linear (Fig. 2 *b*). We therefore developed an ultrasensitive assay to accurately estimate the abundance of PCDH15- and LHFPL5-containing complexes in a mouse cochlea or utricle using SiMoA (38).

**Figure 4:**
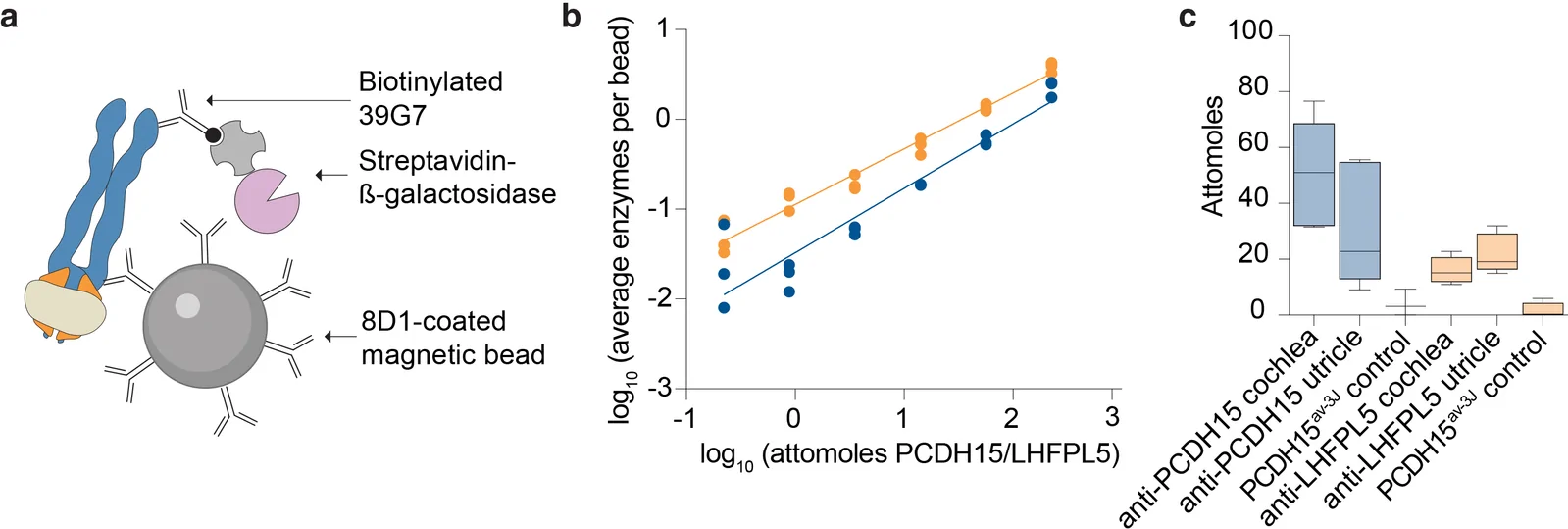
SiMoA assay development and quantification of native PCDH15 and the PCDH15/LHFPL5 complex. (*a*) Schematic depiction of SiMoA capture and detection. PCDH15/LHFPL5 complexes are immobilized on a magnetic bead coated with anti-PCDH15 mAb 8D1. PCDH15 and LHFPL5 are detected with biotinylated anti-PCDH15 mAb 39G7 or anti-LHFPL5 mAb. Streptavidin conjugated to β-galactosidase is used to quantify the number of PCDH15/LHFPL5 complexes per bead. (*b*) Standard curve of recombinant PCDH15 (*blue*) and recombinant LHFPL5 (*orange*). *N* = 3 data points from 3 independent experiments are shown. (*c*) A boxplot depicting quantification of PCDH15 and LHFPL5 isolated from mouse cochlea or utricle. *N* = 6–8 cochlea or utricles for all samples. Box whiskers show the minimum and maximum data values. Cochlea from PCDH15^av−3J^ animals served as a negative control.

In several respects, SiMoA is similar to SiMPull in that MT subunits in cell or tissue lysate are first captured on an antibody-coated surface, which for SiMoA is a paramagnetic bead, and then subsequently detected with a labeled secondary antibody, which in SiMoA is labeled with β-galactosidase (Fig. 4 *a*). Following assembly of immunocomplexes on beads, the beads are loaded into an array of femtoliter-sized reaction wells, each of which can accommodate a single bead, and the β-galactosidase substrate is applied. Beads that possess an enzyme-labeled immunocomplex generate a relatively high local concentration of fluorescent product that is contained within the femtoliter reaction well, thus amplifying the signal and allowing for detection of single complexes.

To develop the SiMoA assay, we used the recombinant PCDH15/LHFPL5 complex and the same antibodies used in SiMPull assays (Fig. 1 *b*). Paramagnetic beads were functionalized with 8D1 and incubated either with recombinant protein sample or, after method optimization, cochlea supernatant prepared identically to SiMPull experiments. The beads were washed extensively and incubated with biotinylated 39G7 or anti-LHFPL5 mAbs, followed by a streptavidin β-galactosidase conjugate (Fig. 4 *a*). The beads were washed again to remove excess streptavidin β-galactosidase and then loaded onto the SR-X Biomarker Detection System. The SR-X is an automated system that works by isolating the beads in arrays of 50-fL reaction wells. Because each well only fits one bead, the number of fluorescent wells corresponds to the number of captured protein complexes. We found we were able to reliably detect 2 amol of recombinant PCDH15 and 4 amol of recombinant LHFPL5 in complex with PCDH15 using this assay, well within the range needed for detection of native MT subunits (Fig. 4 *b*). The standard curve generated using recombinant PCDH15/LHFPL5 was used for quantitation of native proteins in solubilized tissue.

We applied the SiMoA assay to the quantification of native PCDH15 and LHFPL5 from cochlea and utricle supernatant dissected from P6 mice (Fig. 4 *c*). We found an average of 55 amol of PCDH15 per cochlea and 25 amol per utricle. The individual measurements of PCDH15 abundance spanned a wide range, from 31 to 76 amol per cochlea and 9–55 amol per utricle, likely due to experimental variability coupled with variations in mouse development. The number of PCDH15 molecules per stereocilia changes dramatically during hair bundle development up to P9 (45), so measurements of PCDH15 abundance will change due to differences in mouse developmental stage, which can be influenced by litter size and other factors (46,47). A signal above background was not detected for PCDH15 derived from cochlea of PCDH15^av−3J^ animals. The number of PCDH15/LHFPL5 complexes, measured by detecting PCDH15-captured complexes with an anti-LHFPL5 antibody, fell within a similar range, 18 amol per cochlea and 21 amol per utricle. These data suggest that the majority of native PCDH15 is in complex with LHFPL5, in accordance with data obtained from the SiMPull experiments.

## Discussion

We developed SiMPull and SiMoA assays for the detection, quantitation, and characterization of native MT subunits. We applied these assays to the native PCDH15/LHFPL5 complex isolated from mouse cochlea and utricles, demonstrating the exceptional sensitivity and utility of these assays for studying extremely low-abundance proteins. The results of our SiMPull assays indicate the majority of native PCDH15 is bound to LHFPL5 and the two proteins form a stable heterotetrameric complex. SiMoA quantitation reveals an average of 18 amol of PCDH15/LHFPL5 complex and 55 amol of PCDH15 per cochlea, the first quantitative measurement of MT complex subunits.

The SiMPull assay has substantial potential for uncovering the molecular composition and organization of the MT complex, which have eluded scientists for decades. Immunoprecipitation and yeast two-hybrid experiments suggest multiple interactions between MT subunits, but these results are clouded by an inability to reconstitute the complex in heterologous cell lines. The SiMPull assay overcomes this barrier by isolating individual MT subunits from native cochlea and enabling analysis of their stoichiometry through photobleaching experiments, as well as their interactions with other subunits through colocalization experiments. These tools have the potential to elucidate the organization of the MT complex, as well as to report on the distribution of MT complexes if the subunit composition varies. Indeed, a recently developed bead-based SiMPull assay indicates complex formation between TMC1 and LHFPL5, although colocalization and stoichiometry measurements were not possible due to the assay format (48,49).

The SiMoA assay serves as an ideal complement to the SiMPull assay by enabling precise and quantitative measurement of individual MT subunits in the cochlea and utricle. While SiMPull provides valuable information about complex organization and molecular stoichiometry, it is not well suited for quantification due to the small and variably sized assay chamber, which necessitates iterative sample application and introduces potential variability. The measured abundance of PCDH15 and PCDH15/LHFPL5 complex via SiMoA, which ranged from an average of 18 to 55 amol depending on the tissue, is consistent with microscopy experiments that suggest the presence of multiple MT complexes per stereocilium (32,33), rather than a single mature complex at each stereocilia tip. Further, SiMoA is well suited for studying hair bundle assembly, where the abundance of PCDH15 changes significantly during the postnatal period (45). SiMoA could quantify the developmental changes in protein expression, while SiMPull could provide complementary information about changes in protein-protein complexes that accompany the tightly coordinated process of hair bundle assembly (50).

There are, however, several limitations to these methods, the most importance of which is their reliance on high-affinity antibodies. The assays cannot be employed if antibodies with good binding kinetics and affinities do not exist and there is no alternate way to capture the target protein, such as with a genetically engineered tag. Additionally, it is possible that isolation of the complex results in the loss of weakly or transiently bound partners. Although we use gentle solubilization conditions, there is always a possibility that removal of a protein from its native environment disrupts transient interactions. Further, both assays probe the composition of complexes derived from the entire cochlea, not just what is present at the stereocilia membrane. It is possible the functional, fully assembled MT complexes on stereocilia are only a fraction of the total complexes and that differently assembled variants are present as intermediates or alternative species.

## Data and code availability

Custom code used for analyzing single-molecule photobleaching trajectories in this study is available at Zenodo (https://doi.org/10.5281/zenodo.8161179).

## Acknowledgments

The authors thank members of the Gouaux and Baconguis laboratories for helpful discussions and Rachel Courtney for assistance with manuscript preparation. The authors also like to acknowledge the staff in the OHSU Advanced Light Microscopy Core (RRID: SCR_009961), especially Stefanie Petrie, for help with cochlea imaging. This work was supported by 10.13039/100000002NIH grant 1F32DC017894 (to S.C.). E.G. gratefully acknowledges Bernard and Jennifer LaCroute for support. E.G. and T.H. are investigators of the 10.13039/100000011Howard Hughes Medical Institute.

## Author contributions

S.C., J.M., and J.E. performed the experiments. All authors contributed to the design, analysis, and interpretation of experimental data. S.C. and E.G. wrote the manuscript and all authors contributed to the review and editing of the manuscript.

## Declaration of interests

The authors declare no competing interests.

## Supporting material

Document S1. Figures S1–S3

Document S2. Article plus supporting information
