# Supplementary material for: The protocadherin-15-LHFPL5 tip link complex is a heterotetrameric assembly in hair cell stereocilia
Source: Biophys J. 2026 Feb 10;125(6):1464–74. doi: 10.1016/j.bpj.2026.02.003 (PMC13228514; doi:10.1016/j.bpj.2026.02.003)
Supplement: Document S1. Figures S1–S3 [file mmc1.pdf]

Biophysical Journal, Volume *125*

Supplemental information

The protocadherin-15-LHFPL5 tip link complex is a heterotetrameric assembly in hair cell stereocilia

Sarah Clark, Jaba Mitra, Johannes Elferich, April Goehring, Jingpeng Ge, Taekjip Ha, and Eric Gouaux

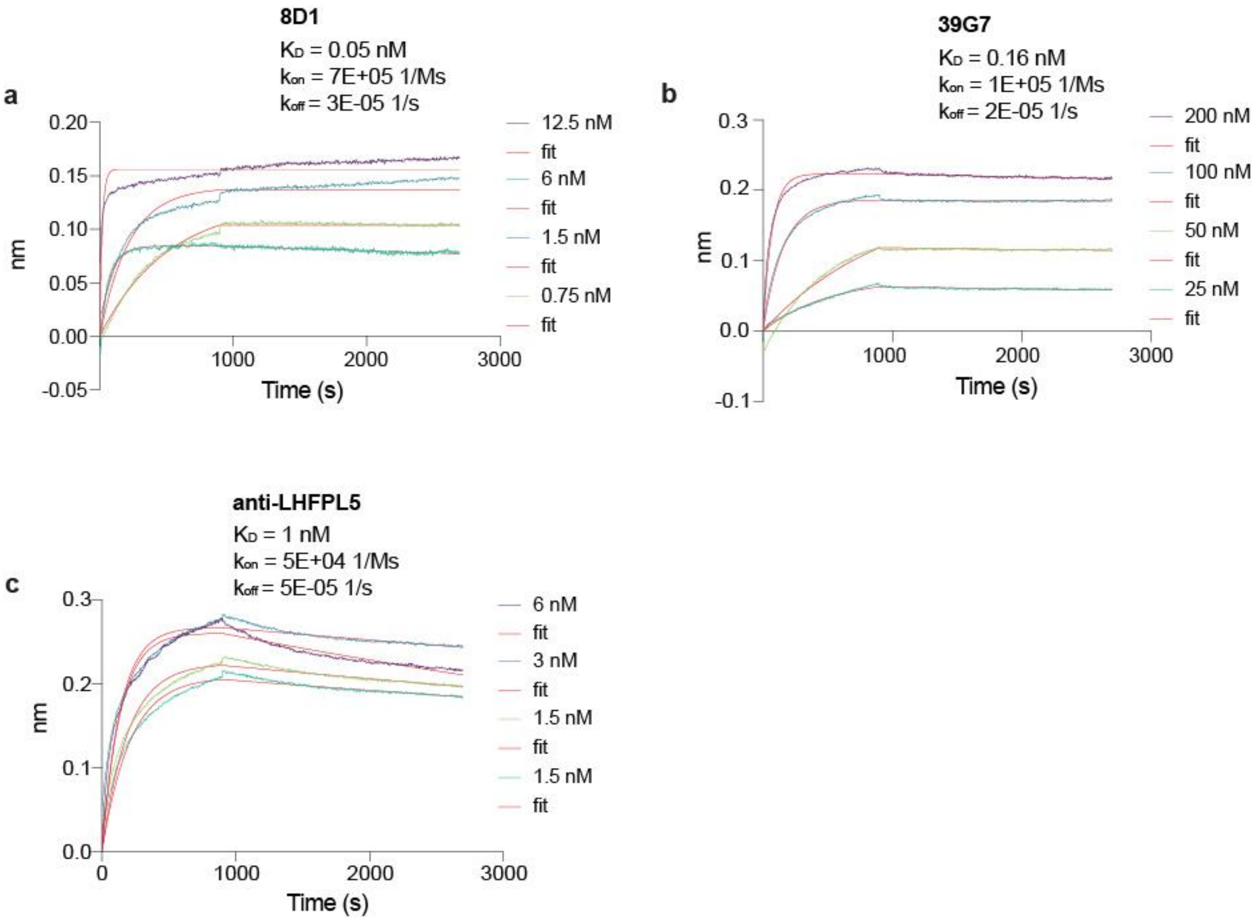

Supplementary Figure 1: Bio-layer interferometry measurements of anti-PCDH15 and anti-LHFPL5 antibodies.

Experimental traces are shown for anti-PCDH15 8D1 **(a),** anti-PCDH15 39G7 **(b),** and anti-LHFPL5 **(c)**. Concentrations of PCDH15/LHFPL5 antigen ranged from 0.75 – 220 nM depending on the experiment.

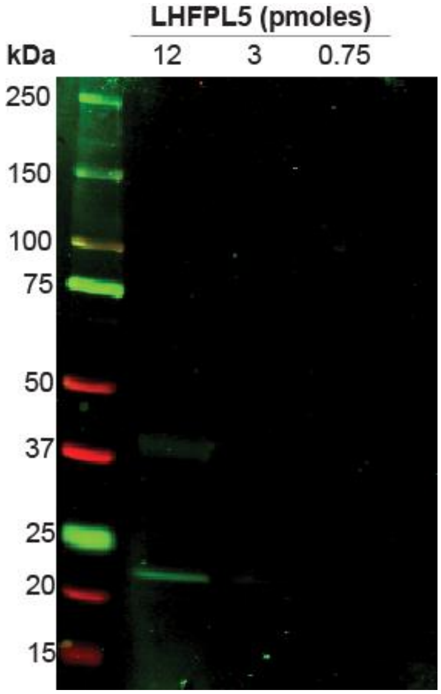

Supplementary Figure 2: Western blot of recombinant LHFPL5.

Recombinant LHFPL5 was probed with anti-LHFPL5 monoclonal antibody at 0.75 pmoles, 3 pmoles, and 12 pmoles, to facilitate direct comparison to SiMPull and SiMoA experiments.

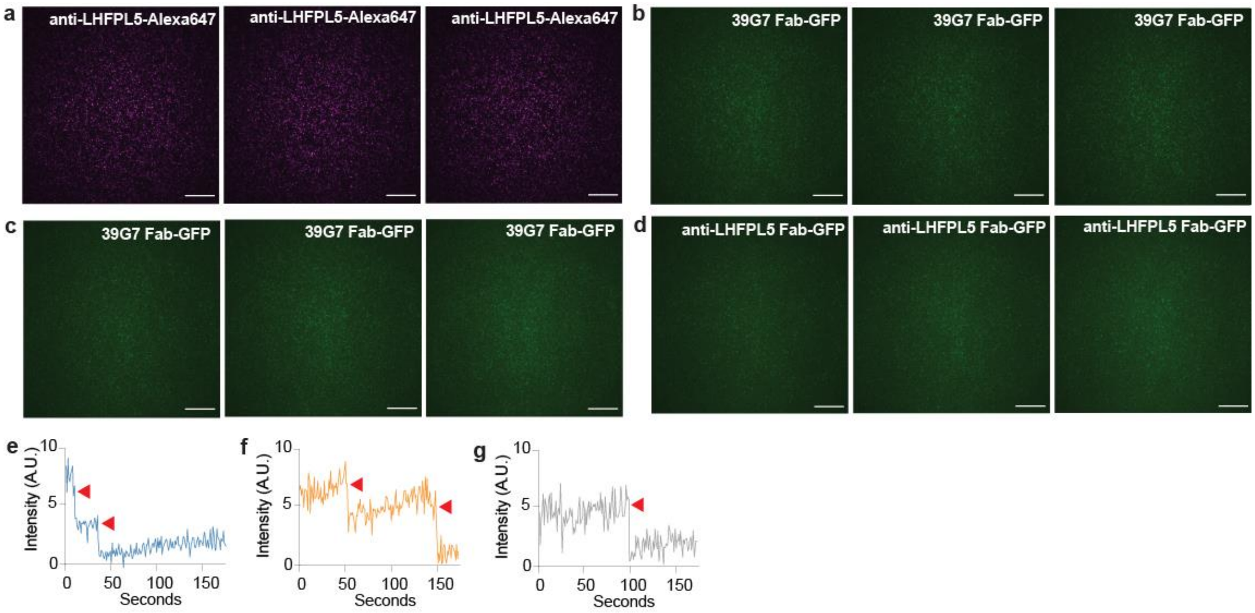

Supplementary Figure 3: Representative raw TIRF images and photobleaching traces for the native PCDH15/LHFPL5 complex captured with the 8D1 antibody.

a-d, Raw images of PCDH15/LHPLF5 from solubilized cochlea captured with the 8D1 mAb and detected with different antibodies or antibody fragments. **a**, Detection with the anti-PCDH15 39G7 Fab-GFP for colocalization experiments. **b,** Detection with the anti-LHFPL5-Alexa647 mAb for colocalization experiments. **c**, Detection with the anti-PCDH15 39G7 Fab-GFP for photobleaching experiments. **d**, Detection with the anti-LHFPL5 Fab-GFP for photobleaching experiments. Scale bars, 20 μm. **e**, Representative trace showing two step photobleaching of 39G7 Fab-GFP that is being used to detect native PCDH15. **f**, Representative trace showing two step photobleaching (red arrows) of anti-LHFPL5 Fab-GFP that is being used to detect native LHFPL5 in complex with PCDH15. **g**, Representative trace showing one step photobleaching (red arrow) of anti-LHFPL5 Fab-GFP.
